# Ovule positions within linear fruit are correlated with nonrandom mating in *Robinia pseudoacacia*

**DOI:** 10.1038/srep36664

**Published:** 2016-11-07

**Authors:** Cunquan Yuan, Yuhan Sun, Peng Sun, Yunfei Li, Ruiyang Hu, Keqi Zhao, Jinxing Wang, Yun Li

**Affiliations:** 1 Beijing Key Laboratory of Ornamental Plants Germplasm Innovation & Molecular Breeding; Beijing Laboratory of Urban and Rural Ecological Environment; National Engineering Research Center for Floriculture, Beijing Forestry University, Beijing, 100083, China; 2 National Engineering Laboratory for Tree Breeding; Key Laboratory of Genetics and Breeding in Forest Trees and Ornamental Plants, Ministry of Education; College of Biological Sciences and Technology, Beijing Forestry University, Beijing, 100083, China; 3 Non-timber Forest Research and Development Center of Chinese Academy of Forestry, Zhengzhou, 450003, China; 4 Wenquan Nursery, Beijing Gardening and Greening Bureau, Beijing, 100095, China

## Abstract

Post-pollination processes can lead to nonrandom mating among compatible pollen donors. Moreover, morphological patterns of ovule development within linear fruits are reportedly nonrandom and depend on ovule position. However, little is known about the relationship between nonrandom mating and ovule position within linear fruit. Here, we combined controlled pollen competition experiments and paternity analyses on *R. pseudoacacia* to better understand nonrandom mating and its connection with ovule position. Molecular determination of siring success showed a significant departure from the expected ratio based on each kind of pollen mixture, suggesting a nonrandom mating. Outcrossed pollen grains, which were strongly favored, produced significantly more progeny than other pollen grains. Paternity analyses further revealed that the distribution of offspring produced by one specific pollen source was also nonrandom within linear fruit. The stylar end, which has a higher probability of maturation, produced a significantly higher number of outcrossed offspring than other offspring, suggesting a correlation between pollen source and ovule position. Our results suggested that a superior ovule position exists within the linear fruit in *R. pseudoacacia*, and the pollen that was strongly favored often preferentially occupies the ovules that were situated in a superior position, which ensured siring success and facilitated nonrandom mating.

More ovules are produced than the number of seeds that mature in most flowering plants. Sometimes, ovules fail to mature seeds due to pollen shortage, leading to eventual abortion^1^,^2^. However, for most flowering plants. pollen quantity is far greater than the ovule number that needs to be fertilized. Thus selection may occur during mating. Naturally, post-pollination processes are considered to be selective in determining the siring success of pollen donors^3^,^4^.

This selection may involve the following cases: discrimination between self and non-self pollen, among compatible donors, between too closely (related) or too distantly related conspecifics, and between interspecific pollen donors^4^,^5^,^6^,^7^. In this case, when more compatible pollen grains are present on a stigma than are needed to fertilize all available ovules, the outcome of mating may be nonrandom because either the pollen donors compete for access to those ovules or because the maternal tissue influences the outcome of mating^4^,^8^,^9^,^10^,^11^. Nonrandom mating has received strong interest for its potential to avoid inbreeding depression and for its potential to be the result of sexual selection^12^,^13^,^14^,^15^,^16^,^17^,^18^,^19^. To better understand nonrandom mating and its implications, pollen competition experiments need to be performed on different species^5^. However, despite a long history of theoretical and experimental attention, little is known about the underlying genetics that govern the process of nonrandom mating^19^,^20^,^21^.

Additionally, ovules ordered linearly within a fruit purportedly differ in their probabilities of reaching maturity. This has been studied for several decades regarding the effect of ovule position on ovule maturation, and nonrandom seed (ovule) abortion and maturation within fruit has been reported in several species^22^,^23^,^24^,^25^,^26^. For example, a study on ovule development patterns in *Bauhinia ungulata* L. showed that non-fertilized and early aborted ovules were often located near the basal end of the ovary; whereas mature seeds were found mainly in the stylar half of fruits where ovules are likely to be fertilized by fast-growing pollen tubes^18^. In contrast, the probability of seed maturation within individual fruits was found to decrease significantly from the basal to stylar end in *Hesperis matronalis* L., suggesting an intra-fruit resource gradient^25^.

Although nonrandom mating and nonrandom patterns of ovule development within linear fruits have long been proposed and studied separately in many species, few attempts have been made to combine studies on the relationship between nonrandom mating and nonrandom ovule development within linear fruit. Our understanding of their internal relationships and inherent influence on siring success remains rudimentary.

Our previous study on ovule developmental patterns in *R. pseudoacacia* from two open-pollinated populations showed that ovule maturation patterns were nonrandom within a fruit^27^. In our other study, selection component and paternity analyses of open-pollinated offspring further indicated that selection existed both within linear fruit and during the early stages of reproduction in *R. pseudoacacia*^28^. Our previous study suggested that selection resulted in an overall decrease in the level of surviving selfed progeny at each life stage (aborted stage, mature seed stage, and seedling stage). Within fruits, the stylar ends showed higher outcrossing rates than the basal ends, indicating selection based on the order of seeds within the fruit^28^,^29^. These results suggest that a connection may exist between nonrandom mating and ovule position in *R. pseudoacacia*.

In this study, we combined controlled pollen competition experiments and paternity analyses on *R. pseudoacacia* to better understand nonrandom mating and its connection with ovule position. We found that a superior position exists within the fruit in *R. pseudoacacia*, and the pollen that was strongly favored in mixed pollen competition often preferentially occupied the ovules that were situated in a superior position, which ensured siring success and facilitated nonrandom mating. These findings may provide novel insights for understanding the selection mechanism of tree species with mixed-mating systems.

## Results

### Ovule maturation patterns are nonrandom within fruit and depend on ovule position

In total, 273 fruits were obtained from different pollination treatments. Each fruit was then divided into four segments (A, B, C and D), beginning at the stylar end and proceeding to the basal end of the fruit. All seeds (ovules) at each position were counted from each fruit. Each seed (ovule) was classified according to its condition as a mature or aborted seed. Ovules that showed no apparent signs of development, or ovules that had expanded in size and had begun some development of the embryo and seed coat, but were necrotic and underdeveloped, were treated as aborted seeds (ovules). Mature seeds were brown-black in color and plump, with a completely formed seed coat, and the seed development pattern followed that reported by Susko^22^. The position of each mature seed within the fruit was recorded. For each fruit and position within the fruit, we calculated the seed abortion rate (number of aborted seeds per fruit or per position/total number of seeds per fruit or per position) and the seed set (number of mature seeds per fruit or per position/total number of seeds per fruit or per position). In total, 1,894 mature seeds were obtained in the four positions from the 273 fruits.

To assess whether ovule maturation patterns were nonrandom within a fruit, ovule development patterns within a fruit were compared. Results showed that the stylar end showed the highest proportion of the mature seed set compared with the lower basal end regardless of the pollination treatments. That is, the abortion rate decreased from the basal end to the stylar end and the mature seed set increased from the basal end to the stylar end (Table 1). These results are consistent with our previous report, and Susko’s results in open-pollinated *R. pseudoacacia*^22^,^27^, indicating a nonrandom ovule development pattern depending on the position of the ovule within the fruit in *R. pseudoacacia*.

### Siring success is nonrandom among compatible pollen donors

To test whether siring success was nonrandom among compatible pollen donors in mature seed and seedling staged, mature seeds obtained from different controlled competitive pollination treatments were randomly divided in half; one half was used to grow seedlings, and then the paternity of each offspring (mature seeds and seedlings) was assigned by paternity analysis.

The genetic variation of the six microsatellite loci used in the paternity analysis was large among the samples (Supp. Table S1, Supp. Figure S1). The total exclusion probability for the second parent, corresponding to the total exclusionary power for the pollen parent, was 0.9946, which allowed precise paternity analysis (Supp. Table S2).

**Figure 1:**
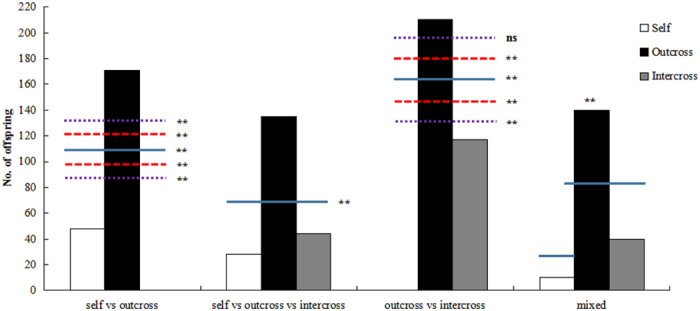
Number of mature seed offspring produced by each pollen source in competition experiments. Blue solid line indicates pollen mixtures of 1:1, while the long red dashed line indicates pollen mixtures with 10% bias, and the short purple dashed line indicates pollen mixtures with 20% bias. The single asterisk indicates statistically significant differences relative to the expected pollen mixture ratio (p < 0.05), the double asterisk indicates statistically significant differences relative to the expected pollen mixture ratio (p < 0.01), and ns indicates no significant difference.

In mature seed offspring, paternity analysis revealed that the observed proportions of offspring obtained from the two donor competitive pollinations were significantly different from the expected pollen ratio of 1:1. Indeed, 3.56 times more offspring were produced by outcross-pollen donors than by self-pollen donors in 1:1 pollen of self versus outcross pollination. Moreover, 1.79 times more offspring were produced by outcross-pollen donors than by intercross pollen donors in 1:1 pollen of outcross versus intercross pollination (Supp. Table S3). Because pollen mixtures may not occur in a precise 1:1 ratio, a set of tests with expectations of 0.55:0.45 and 0.6:0.4 were also performed on data from the two donor pollinations. Thus, these 0.55:0.45 and 0.6:0.4 expectations tested the validity of our findings even if our pollen mixtures were 10–20%-biased. χ^2^ tests showed that the bias toward selfed offspring was also significant if self to outcross pollen ratios had consistently been 10–20% different (0.55:0.45 and 0.6:0.4 expectations, respectively). The χ^2^ tests showed that the bias toward siring offspring (1:1) was also significant when the cross to intercross pollen ratio was consistently 10% different from that expected (0.55:0.45 expectations; Fig. 1). Paternity analysis also revealed that the observed frequencies of self, cross, and intercross offspring were significantly different from the expected ratio in three donor pollination and mixed donor pollination cases (Fig. 1). Most offspring were produced via outcross fertilizations, which were 4.82 times more common than self-fertilizations and 3.07 times more common than intercross fertilizations (Supp. Table S3).

**Figure 2:**
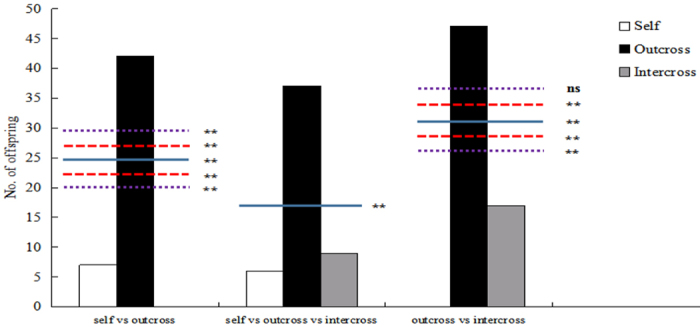
Number of seedling offspring produced by each pollen source in competition experiments.

In seedling offspring, paternity analyses also revealed that the observed proportions of offspring were significantly different from the expected pollen ratio. Outcrossed pollen grains were strongly favored and produced significantly more progeny than other pollen grains (Fig. 2). Six times more seedlings were produced by outcross-pollen donors than by self-pollen donors in a 1:1 pollination. Cross pollen donors produced 2.8 times more seedlings than intercross pollen donors. In three-donor pollinations, most offspring were produced via outcross fertilizations, which were 6.1 times more common than self-fertilizations and 4.1 times more common than intercross fertilizations.

The results above suggested that siring success was nonrandom among compatible pollen donors in *R. pseudoacacia*. Outcrossed pollen grains, which were strongly favored, produced significantly more progeny than other pollen grains.

### Offspring produced by one specific pollen source are nonrandomly distributed within the four position of a linear fruit

**Figure 3:**
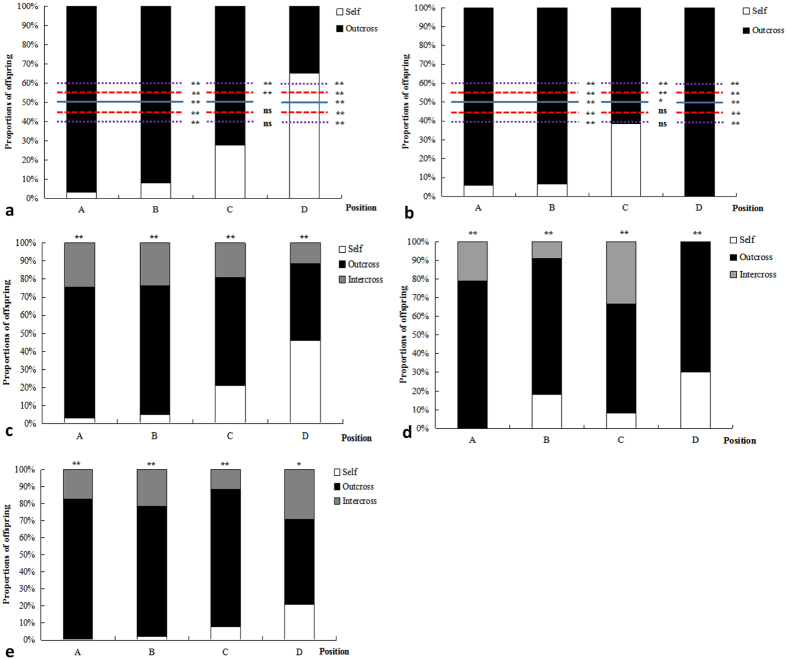
Percentage of total offspring in each position that the specific pollen source produced in competitive pollinations at the mature seed and seedling stages. (**a**) Self versus outcross (pollen mixture of self:outcross = 1:1) at the mature seed stage, (**b**) Self versus outcross at the seedling stage, (**c**). Self versus outcross versus intercross (pollen mixture of self:outcross:intercross = 1:1:1) at the mature seed stage; (**d**). Self versus outcross versus intercross at the seedling stage; (**e**). Mixed pollination (pollen mixture of self:outcross:intercross = 1:3:3) at the mature seed stage. In position A, the observed proportion of outcrossed offspring was significantly higher than the competitors, regardless of the competitive pollination treatment.

To further test whether siring success was correlated with pollen source and ovule position, paternity analyses were conducted among the four positions within the fruit. Here, χ^2^ tests were used to determine whether the offspring produced by a specific pollen source were distributed randomly at each position. The results showed that the offspring produced by one specific pollen source were not randomly or equally distributed within the four positions of the linear fruit. Within the linear fruit, the observed proportions of offspring at each position were significantly different from the expected pollen ratio, even with 10–20%-biases. At the stylar end (position A), which had a higher probability of maturation, outcrossed pollen produced a significantly higher proportion of outcrossed offspring than any other pollen source in both mature seed offspring and seedling offspring. Whereas the proportion of outcrossed offspring decreased from the stylar end to the basal end (from positions A to D), the proportion of offspring produced by less competitive pollen sources increased from positions A to D (Fig. 3, Supp. Tables S4–S6).

Taken together, these results indicate that the distribution of offspring produced by one specific pollen source was nonrandom within the fruit, but dependent on both pollen donor and ovule position.

## Discussion

Post-pollination processes are considered to be a selective agent in determining the siring success of pollen donors. Our results showed that ovule maturation patterns are nonrandom within a linear fruit and depend on ovule position in *R. pseudoacacia*. Moreover, post-pollination mechanisms in *R. pseudoacacia* led to nonrandom mating among compatible pollen donors. Siring success favored outcross pollen and strongly rejected self-pollen. Our results also showed that the distribution of offspring produced by one specific pollen source was nonrandom within a fruit, but dependent on pollen donor and ovule position. From our results, we conclude that ovule positions within a linear fruit are correlated with nonrandom mating in *R. pseudoacacia.* Within a linear fruit, the strongest pollen competitors have the best chances of fertilizing ovules that are situated in a superior position, which ensures development into mature seeds. This mechanism contributes to the post-pollination mechanisms that ultimately determine the outcome of mating and finally lead to nonrandom mating.

### Nonrandom mating in controlled competitive pollination

In this study, outcrossed pollen grains were strongly favored regardless of the pollination treatment. In both pollinations, outcrossed pollen grains showed a significantly higher number of offspring than the other pollen grains at both the mature seed and seedling stages.

The higher success rate of outcrossed pollen at siring offspring versus self-pollen may be a consequence of the pollen tube growth rate being higher for outcross pollen than for self-pollen. In our previous study, it showed that outcross pollen germinates and grows faster than self-pollen in the pistil and style of *R. pseudoacacia*^27^. Based on this, we speculate that in self + outcross pollen mixtures, outcross pollen grains have a significant fertilization advantage. Thus, different pollen tube growth rates of self- and outcross-pollen grains may function as a post-pollination mechanism in pollen mixtures. This phenomenon has also been recorded for other self-compatible species with floral traits typical of outcrossing mating systems^18^,^30^,^31^,^32^,^33^,^34^.

However, pollen tube growth rate might not be the only mechanism determining the siring success of pollen donors. Other pre-zygotic mechanisms such as interactions between pollen grains and style may also play roles in this process. In self-cross versus outcross versus intercross (1:1:1) pollinations, outcross seedlings occurred 6.1 times more often than self-cross fertilizations and 4.1 times more often than intercross fertilizations, and these values were 4.82 and 3.07 times, respectively, in mature seed offspring. In a previous study, our research team reported that self and outcrossed pollen grains can germinated and pollen tubes can passed successfully through the styles^27^,^35^. However, when germinated in the stigma of *R. pseudoacacia*, only a few pollen tubes of *Robinia pseudoacacia* var. *decaisneana* (Carr.) Vos reached the ovary tissue, twisted pollen tubes were observed in the style of *R. pseudoacacia*^35^. It has been documented in other studies that interactions between pollen grains and style may favor or prevent germination rate, and tube attrition, and thus inhibit or facilitate pollen tube growth when growing through^18^,^30^,^31^,^32^,^34^,^36^,^37^. Therefore, this mechanism may have favored the greater success of outcross pollen at siring success in *R. pseudoacacia*. Moreover, the delivery of mixed pollen loads to the stigmatic surface of plants can lead to gametophytic selection^38^.

In addition, post-zygotic mechanisms such as seed abortion, fruit abortion, seed filling and seed germination may also determine the siring success of pollen donors^1^,^6^,^18^,^39^,^40^,^41^,^42^,^43^,^44^,^45^. Inbreeding depression is probably a strong selection force that acts on the siring success of offspring in *R. pseudoacacia*. In our previous study, progenies resulting from cross-pollination treatments showed significantly higher fitness than progenies resulting from self-pollination, which exhibit high levels of inbreeding depression. The highest inbreeding depression was observed between the fertilization and seed maturation stages, whereas the seedling emergence stage showed the second highest level of inbreeding depression^29^. In the present study, outcross-pollen donors produced six times more seedlings than self-pollen donors in the 1:1 pollen mixtures. Combined with our previous study, our conclusion is that when self- and outcross-pollen grains of *R. pseudoacacia* were present on the pistil simultaneously in the pollen mixture, outcross pollen took precedence over self-pollen in fertilizing ovules due to more rapid pollen tube growth. Once ovules were fertilized by self-pollen, some of them may be aborted due to early inbreeding depression. Although we did not determine the genotype of aborted seeds in either self- or outcross-pollen mixtures, we genotyped the aborted seeds of *R. pseudoacacia* in open pollination, and results showed that the aborted seeds exhibited a slight heterozygote deficit and showed high levels of inbreeding^28^. If some ovules fertilized by self-pollen in the pollen mixture succeed in maturing, some of them may not germinate, thus reducing the number of selfed offspring even further.

Taken together, all of the above post-pollination mechanisms might result in nonrandom mating, especially the higher rate of siring success in outcross seedlings from pollen mixtures. However, we cannot rule out other factors also acting on post-pollination mechanisms.

### Nonrandom patterns of ovule maturation and abortion within linear fruit

When dividing ovule production into different positions within fruit for analysis of the pollination results, we observed that the stylar end showed a significantly higher mature seed set, but a lower abortion rate compared to the middle or basal ends, indicating the existence of advantageous ovule position within fruit. This is consistent with our previous observations of open-pollinated fruit in which fertilization gradients exist^27^. The effect of ovule position on seed maturation patterns and abortion in plants has been studied for several decades, and nonrandom seed (ovule) abortion and maturation within fruit has been characterized in many species with open pollination^22^,^23^,^24^,^25^,^26^. In *H. matronalis*, the probability of seed maturation within individual fruits decreased significantly from the basal to stylar ends, suggesting an intra-fruit resource gradient^25^, whereas in *B. ungulata*, mature seeds were found mainly in the stylar half of fruits, where ovules are likely to be fertilized by quickly growing pollen tubes, suggesting a fertilization gradient^23^.

Several hypotheses have been proposed to explain the high levels of seed abortion and nonrandom abortion patterns in most plant species. Some argue that many seeds may be aborted due to the expression of lethal or deleterious alleles, and a high level of homozygosity due to inbreeding may lead to high seed abortion rates. Under these conditions, abortion may be random within fruit^26^. In outcrossing species, seed abortion increases as the level of inbreeding increases as a result of the genetic load.

Others argue that abortion depends on the supply of resources for seed development^46^. Ovules situated nearest the base of a fruit have an inherent spatial advantage when competing r resources with more stylarly located ovules because ovules nearer to the base of a fruit are in closer physical proximity to the vascular supply tissues of the pedicel. Assuming that all the ovules are fertilized, the zygotes that are closer to the source of resources will receive resources first and/or in larger quantities than zygotes farther from the source. According to this hypothesis, the ovules (zygotes) at the basal ends are more likely to develop into mature seeds. In contrast, ovules at the stylar ends are likely to abort since they are farther from the supply of resources^46^,^47^.

Additionally, others still argue that seed abortion may be due to differences in the ability of developing seeds to gather maternal resources^48^,^49^. Based on this hypothesis, all seeds within a fruit must compete for resources allocated by the maternal plant, and stronger seeds may more efficiently obtain resources than less competent seeds (which will ultimately starve). It has been reported that offspring vigor is related to the vigor of the paternal plant^48^,^50^. Therefore, stronger, faster-growing pollen tubes will give rise to stronger seeds. Under these conditions, seed abortion is the consequence of gametophytic competition for access to ovules^49^.

Combining the above hypothesis with our pollination results in this report, we assumed that the higher probability of ovule (seed) maturation at the stylar ends is due to the closer proximity to the point of entry of pollen tubes, while those at higher probability of ovule (seed) abortion in the basal ends are due to being located farther from the entry point. We further considered that the early fertilization of stylar ovules and the failure of basal ovules to develop into mature seeds are attributable to fast-growing pollen tubes and the early initiation of stylar ovules, which provides a temporal “head start” and allows ovules to develop into stronger regional sinks for resources.

### Relationship between nonrandom mating and patterns of ovule development within fruit

In the above section, we discussed that the higher success at siring offspring of outcross pollen compared to self-pollen might be a consequence of the pollen tube growth rate being higher for outcross pollen. In this situation, we suggest that the pollen with a faster germination rate and pollen tube growth rate would preferentially fertilize the stylar ovules in mixed pollination to increase the probability of maturation. Paternity analysis of offspring from the four positions within a fruit in mixed pollination showed that the distribution of offspring produced by one specific pollen source was not random within linear fruit. The number of offspring produced by outcross pollen decreased from stylar end to basal end. Instead, the stylar end showed the lowest number of offspring produced by self-pollen, suggesting a correlation between the pollen source and ovule position. In our previous study of open-pollinated *R. pseudoacacia* offspring, we found that the rate of selfed progeny originating from the basal ends (0.6169) was higher than their origin at the stylar ends (0.3831)^27^. The results above further support our hypothesis.

As discussed above, in our examination of pollen tube growth from self, outcross and intercross pollen, the intercross-pollen tube grew the fastest, followed by the outcross-, with the self-pollen tube growing the most slowly. However, a significantly higher number of offspring produced by outcrossed pollen than intercrossed pollen in competitive pollination suggested that other mechanisms, such as outbreeding depression, may be involved in determining the siring success of pollen donors in *R. pseudoacacia*.

Our previous study showed high levels of inbreeding depression in *R. pseudoacacia*^28^, which further reduced the selfed offspring in competitive pollination, combined with the disadvantage of self-pollen in the fertilization gradient within fruit. Because we did not determine the paternity of aborted seeds in competitive pollination, we could not ascertain whether the ovules fertilized by intercross pollen were aborted. We also could not determine whether, or how many, mature seeds fertilized by intercross pollen could not germinate. However, the fact that the number of offspring produced by intercross-pollen decreased from the stylar end to the basal end suggested that the position effect also plays a role in competitive pollination with intercross hybridization in *R. pseudoacacia*.

## Conclusions

Nonrandom mating has received theoretical and experimental attention for its role as a consequence of inbreeding depression and sexual selection as well as for its role in reinforcing reproductive barriers and speciation. Furthermore, ovules linearly ordered within a fruit have been claimed to differ in their probabilities of reaching maturity. However, much less attention has been paid to the connection between nonrandom mating and nonrandom patterns of ovule maturation based on ovule position within linear fruit. Here, we showed that the post-pollination mechanism determined the siring success of different pollen donors in *R. pseudoacacia.* The nonrandom distribution of offspring produced by one specific pollen source in mixed competitive pollination within fruit suggested that ovule position effects contribute to these mechanisms that ultimately determine the production of offspring in *R. pseudoacacia*. Our results implied that the pollen source that was strongly favored in mixed pollen competition often preferentially occupies the ovules that were situated in superior position, which ensured siring success and facilitated nonrandom mating. Our research combined the controlled pollination with paternity analysis and provided evidence that siring success is the consequence of both pollen source and ovule positional effects.

## Materials and Methods

### Plant materials and study site

Field-controlled pollination experiments were carried out at the Mijiabu Tree Farm in Yanqing Country, Beijing (40°30′302″N, 116°00′015″E), China, in 2011, 2012, and 2013. Four *R. pseudoacacia* trees and three *Robinia pseudoacacia* var. *decaisneana* (Carr.) Vos trees were selected for manual pollination in this study. The four *R. pseudoacacia* trees (plants A–D) were treated as both pollen receptors (maternal trees) and pollen donors (paternal trees). Pollen collected from the four *R. pseudoacacia* maternal trees was treated as either a self- or outcross-pollen source within species. The three *Robinia pseudoacacia* var. *decaisneana* (Carr.) Vos trees (plants 1–3) were treated as pollen donors. Pollen collected from three *Robinia pseudoacacia* var. *decaisneana* (Carr.) Vos trees was treated as an interspecific pollen source. All the pollen grains used in this study were confirmed as compatible donors, and the pollination combinations used in this study had been shown to produce seeds successfully in preliminary experiments.

### Pollen collection and quantification

Pollen was collected by gently tapping the anthers over a Petri dish, after which the pollen grains were mixed according to preset combinations and proportions. Subsequently, the pollen grains were put into a centrifuge tube and stored at 4 °C for use in pollinations.

### Controlled pollination experiments

We designed the following three pollination combinations: two-pollen-donor, three-pollen-donor, and multiple-mixed-pollen-donor pollinations. In all cases, the four *R. pseudoacacia* plants also acted as pollen donors within species (self- and/or outcross-pollen donors). Each maternal plant was crossed with three different pollen donors. For self- versus outcross-pollen crosses, each maternal plant was crossed with its own pollen and three other maternal pollen grains from different plants (Supp. Table S7a). For outcross- versus interspecific-pollen crosses, all four maternal plants acted as outcross pollen donors, whereas plants from three *R. pseudoacacia* var. *decaisneana* plants were used as donors of interspecific pollen (Supp. Table S7b). Three-donor crosses were conducted in a similar way, and included self pollen, outcross pollen, and interspecific pollen (Supp. Table S7c). For multiple-mixed-pollen-donor pollinations, pollen from four maternal plants and the other three interspecific plants were mixed in an equal ratio (Supp. Table S7d).

In two-pollen-donor pollinations, self pollen and outcross pollen were applied at a ~1:1 ratio for self versus intraspecific pollen crosses. Outcross pollen and intercross pollen were also applied at a ~1:1 ratio for intraspecific versus interspecific crosses. In three-pollen-donor pollinations, self pollen, outcross pollen, and intercross pollen were applied at a ~1:1:1 ratio for the pollination of maternal trees. Pollen grains were also mixed in an equal ratio for each pollen in multiple-mixed-donor pollinations.

According to the experiment design above, 10 different kinds of crosses for each maternal plant were made: three kinds of self versus outcross two-donor crosses, three kinds of outcross versus intercross two-donor crosses, three kinds of self versus outcross versus intercross three-donor crosses, and one multiple-mixed-donor cross. In total, 100 flowers were pollinated for one kind of cross per maternal plant, and, in total, 4,000 flowers were pollinated (4,000 = 4 maternal plants × 10 different kinds of crosses per each maternal tree × 100 flowers per crosses per maternal tree).

At 24 h before anther dehiscence, flowers were emasculated and bagged^29^. Mixed pollen was applied to the target stigma with cotton swabs at 8AM, when the stigma was at optimal receptivity^27^,^29^. Nets were removed 1 week after pollination to return flowers to natural illumination and ambient temperature conditions. Mature fruits were collected before seed dispersal.

### Fruit characteristics and seed set

*R. pseudoacacia* falls within the Fabaceae; fruits are typical legumes with an average of “9–26” linearly arranged ovules, arranged alternately along the suture and that retain a persistent connection to the ovarian wall. Thus, it is possible to record the relative position and development patterns of each ovule.

For each fruit, length, width, and weight were measured with electronic digital calipers and a sensitive balance. Each fruit was then divided into four segments (A, B, C, and D), beginning at the stylar end and proceeding to the basal end. All seeds (ovules) at each position were counted for each fruit. Each seed (ovule) was classified according to its condition, as a mature or aborted seed. Ovules that showed no apparent sign of development, or ovules that had expanded in size and had begun some development of the embryo and seed coat, but were necrotic and underdeveloped, were treated as aborted seeds (ovules). Mature seeds were brown-black in color and plump, with a completely formed seed coat, and the seed development pattern followed that reported by Susko^22^. The position of each seed within the fruit was recorded. For each fruit and position within the fruit, we calculated the seed abortion rate (number of aborted seeds per fruit or per position/total number of seeds per fruit or per position) and the seed set (number of mature seeds per fruit or per position/total number of seeds per fruit or per position) to test for differences among pollination treatments and positions.

Mature seeds per position from the same kind of crosses per maternal tree were divided randomly into two equal parts. One was used for seed paternity analysis and the other was germinated for seedling paternity analysis.

### Germination experiment

Mature seeds were germinated in a phytotron and used to determine seedling paternity. The seeds were placed on moist filter paper in Petri dishes, and then hot water (90 °C) was added and the seeds were soaked for 24 h. The seeds were then placed on new moist filter paper in Petri dishes for another 48 h and then planted into a seedling pot (diameter = 12 cm, height = 12 cm) containing potting soil (roseite:turfy soil:sand:perlite = 2:2:2:1). Seedlings were grown under the following conditions: 14 h of daylight and 10 h of night, 25 °C during the day and 15 °C at night, a daytime lumen output of 40 μmol·m^−2^·s^−1^, and a humidity of 70%. DNA was extracted from leaves as soon as seedlings had enough vegetative tissue to take samples (~4 weeks after seed planting).

### DNA extraction and genotyping

Mature seeds were first ground in microcentrifuge tubes and soaked in pre-treatment solution (100 mmol/L Tris-HCl (pH = 8.0), 100 mmol/L EDTA (pH = 8.0), 2% PVP, 2% β-mercaptoethanol) for 24 h. Then, total DNA from the seed tissues was isolated in the same way as with leaf tissues of parents and seedlings using the Plant DNA Extraction Kit (Tiangen Biotech, Beijing, China) according to the manufacturer’s protocol.

Six simple sequence repeat (SSR) primers were selected and labeled with a fluorescent dye for the paternity analyses (Supp. Table S6)^51^,^52^,^53^. Polymerase chain reaction (PCR) amplification was performed using an ABI 9700 thermal cycler (Applied Biosystems, Foster City, CA, USA) in a 12.5-μL reaction mixture containing 30 ng of genomic DNA, 1 × Taq buffer, 2.5 mM MgCl_2_, 0.25 mM dNTPs, 0.20 μM of each primer pair, and 0.5 units of Taq DNA polymerase. The amplification reaction was performed with the following two procedures: an initial denaturation step at 94 °C for 5 min, followed by 10 cycles at 94 °C for 30 s, melting temperature (T_m_) for 30 s (decreasing at n°C per cycle, n = (T_mmax_ − T_mmin_)/10), 72 °C for 90 s, 20 cycles at the annealing temperature, and a final extension at 72 °C for 10 min, and an initial denaturation step at 94 °C for 5 min, followed by 35 cycles at 94 °C for 30 s, T_m_ for 30 s, and 72 °C for 90 s, followed by a final extension at 72 °C for 10 min.

PCR products were electrophoresed using an ABI 3100 Genetic Analyzer (Applied Biosystems). Allele sizes were determined using the fragment analysis software packages Genescan 3.0 and Genotyper 2.1 (Applied Biosystems).

### Paternity analysis

The paternity of each offspring (mature seeds and seedlings) was assigned by comparing the genotypes of adults and offspring with the computer program CERVUS 3.0^54^, based on a maximum-likelihood paternity assignment. The simulation parameters for CERVUS were as follows: 10,000 tests were performed, 88% of the candidate parents were sampled, and 100% of the loci were typed. A typing error rate of 0% and a confidence level of 80% were used.

### Statistical analyses

We analyzed the effects of pollination treatment on seed set and abortion rate using a one-way analysis of variance. Also, χ^2^ tests were used to determine if the number of offspring (both mature seed and seedling) produced by different pollen sources was nonrandom, i.e. as expected based on the pollen mixtures applied to the stigmas, and if the number of offspring produced by different pollen sources in the same position within fruit was nonrandom. The following χ^2^ tests were made. First, we tested whether the offspring produced by different sources of pollen (self vs. outcross, outcross vs. interspecific, and self vs. outcross vs. interspecific) were significantly different from the expected ratios of 1:1 or 1:1:1, for the two- and three-donor pollinations, respectively. Because pollen mixtures may not occur in a precise 1:1 ratio, sets of tests with expectations of 0.55:0.45 and 0.6:0.4 were also performed on data from the two-donor pollinations. Thus, the 0.55:0.45 and 0.6:0.4 expectations tested the validity of our findings even if our pollen mixtures were 10–20% biased. Second, we tested whether offspring produced by different pollen sources were significantly different from the expected ratios (1:3:3) for the multiple-mixed-donor pollinations. Third, χ^2^ tests were used to determine whether the number of offspring produced by different pollen sources in the same position within fruit was nonrandom: that is, whether the proportions of offspring produced by different pollen sources (self, cross, intercross) were significantly different from the 1:1 ratio (two-pollen donor) or 1:1:1 ratio (three-pollen donor). Moreover, 10–20% biases were also assessed with χ^2^ tests. All analyses were performed using the SPSS software (ver. 16.0; SPSS Inc., Chicago, IL, USA).

## Additional Information

**How to cite this article**: Yuan, C. *et al*. Ovule positions within linear fruit are correlated with nonrandom mating in *Robinia pseudoacacia.* *Sci. Rep.* **6**, 36664; doi: 10.1038/srep36664 (2016).

**Publisher’s note:** Springer Nature remains neutral with regard to jurisdictional claims in published maps and institutional affiliations.

## Supplementary Material

Supplementary Information

## Figures and Tables

**Table 1:** Mature seed sets of different positions within the fruit from different pollen competition experiments.

Competitive pollination combination	**Mature seed rate**				
	Position A	Position B	Position C	Position D	*P*-value
Self versus Outcross	0.3804 ± 0.0462b	0.3915 ± 0.0507b	0.3079 ± 0.0430b	0.1256 ± 0.0241a	<0.0001
Self versus Outcross versus Intercross	0.5169 ± 0.0573c	0.4163 ± 0.0499bc	0.3240 ± 0.0458b	0.1039 ± 0.0259a	<0.0001
Outcross versus Intercross	0.4372 ± 0.0434b	0.4655 ± 0.0455b	0.3797 ± 0.0423b	0.1197 ± 0.0270a	<0.0001
Mixed	0.5317 ± 0.0588b	0.4529 ± 0.0590b	0.4653 ± 0.0580b	0.1568 ± 0.0391a	<0.0001

Average values followed by the same letter were not significantly different (Tukey’s multiple comparison test, *P* ≤ 0.05).
