# Supplementary material for: Ovule positions within linear fruit are correlated with nonrandom mating in Robinia pseudoacacia
Source: Sci Rep. 2016 Nov 7;6:36664. doi: 10.1038/srep36664 (PMC5098198; doi:10.1038/srep36664)
Supplement: Supplementary Information [file srep36664-s1.pdf]

Ovule positions within linear fruit are correlated with nonrandom mating in *Robinia pseudoacacia*

Cunquan Yuan[1, 2], Yuhan Sun[2], Peng Sun[2, 4], Yunfei Li[3], Ruiyang Hu[2], Keqi Zhao[2], Jinxing Wang[2] & Yun Li[2]

[1]National Engineering Research Center for Floriculture, Beijing Forestry University, Beijing 100083, China
[2]National Engineering Laboratory for Tree Breeding; Key Laboratory of Genetics and Breeding in Forest Trees and Ornamental Plants, Ministry of Education; College of Biological Sciences and Technology, Beijing Forestry University, Beijing 100083, China
[3]Wenquan Nursery, Beijing Gardening and Greening Bureau, Beijing 100095, China
[4]Non-timber Forest Research and Development Center of Chinese Academy of Forestry, Zhengzhou 450003, China

Correspondence should be addressed to Yun Li (Email: yunli@bjfu.edu.cn).

Supporting Information

Additional supporting information may be found in the online version of this article.

Table S1 Characteristics of six microsatellite loci in the analyzed population

**Locus**	**Number of alleles**	**N**	H_O	H_E	***PIC***
Rops06	10	1009	0.935	0.751	0.713
RP206	6	1061	0.450	0.640	0.577
Rops08	8	1011	0.546	0.780	0.746
RP109	11	1059	0.773	0.792	0.762
Rops05	11	1061	0.786	0.702	0.680
RP200	18	1056	0.896	0.889	0.879
Average	10.67	1043	0.731	0.759	0.726

Table S2 Exclusion probability of six microsatellite markers used for the paternity analysis

		Exclusion probability				
**Locus**	**Number of alleles**	**First parent**	**Second parent**	**Parent pair**	**Identity**	**Sib identity**
Rops06	10	0.356	0.533	0.721	0.900	0.600
RP206	6	0.221	0.376	0.544	0.808	0.522
Rops08	8	0.394	0.572	0.755	0.918	0.619
RP109	11	0.420	0.597	0.782	0.927	0.627
Rops05	11	0.324	0.514	0.728	0.889	0.573
RP200	18	0.636	0.777	0.924	0.978	0.689
Average	10.67	0.3918	0.5615	0.7423	0.9033	0.6050
Total	64	0.9566	0.9946	0.9999	0.9999	0.9964

Table S3. Offspring sired by each pollen source in competition experiments at the mature seed stage

	**Self**	**Outcross**	**Intercross**
**Self versus Outcross (1:1)**	48	171	
**Self versus Outcross versus Intercross (1:1:1)**	28	135	44
**Outcross versus Intercross (1:1)**		210	117
**Mixed (1:3:3)**	10	144	40

Table S4. Percentage of total offspring in each position that the specific pollen source sired in self versus outcross competitive pollination at the mature seed and seedling stages

	**Mature seed**		**Seedling**	
**Position**	**Self (%)**	**Outcross (%)**	**Self (%)**	**Outcross (%)**
**A**	3.23	96.77	5.88	94.12
**B**	7.94	92.06	6.67	93.33
**C**	27.78	72.22	38.46	61.54
**D**	65	35	0	100

Table S5. Percentage of total offspring in each position that the specific pollen source sired in self versus outcross versus intercross competitive pollination at the mature seed and seedling stages

	**Mature seed**			**Seedling**		
**Position**	**Self(%)**	**Outcross(%)**	**Intercross(%)**	**Self(%)**	**Outcross(%)**	**Intercross(%)**
**A**	2.86	72.86	24.28	0	78.95	21.05
**B**	5.08	71.19	23.73	18.18	72.73	9.09
**C**	21.15	59.62	19.23	8.33	58.33	33.34
**D**	46.15	42.31	11.54	30	70	0

Table S6. Percentage of total offspring in each position that the specific pollen source sired in mixed competitive pollination at the mature seed stage

	**Mature seed**		
**Position**	**Self(%)**	**Outcross(%)**	**Intercross(%)**
**A**	0	82.81	17.19
**B**	1.96	76.47	21.57
**C**	7.27	72.73	10.20
**D**	20.83	50	29.17

Table S7. Pollination combination designs

a. Self versus outcross pollen

Maternal plant	***Pollen donors***		
A	A+B	A+C	A+D
B	B+A	B+C	B+D
C	C+A	C+B	C+D
D	D+A	D+B	D+C

b. Outcross versus interspecific pollen

Maternal plant	***Pollen donors***		
A	B+1	C+2	D+3
B	A+3	C+1	D+2
C	A+2	B+3	D+1
D	A+1	B+2	C+3

c. Self versus outcross versus interspecific pollen

Maternal plant	***Pollen donors***		
A	A+B+1	A+C+2	A+D+3
B	B+A+3	B+C+1	B+D+2
C	C+A+2	C+B+3	C+D+1
D	D+A+1	D+B+2	D+C+3

d. Mixed pollen

Maternal plant	***Pollen donors***
A	A+B+C+D+1+2+3
B	A+B+C+D+1+2+3
C	A+B+C+D+1+2+3
D	A+B+C+D+1+2+3

Note: *Letters* indicate pollen donors within the species, whereas *numbers* correspond to interspecies pollen donors.

Table S8. Primer sequences for six microsatellites of *Robinia pseudoacacia* with fluorescent labeling

Locus	Fluorescent labeling	Motif	Primer sequence	Size range (bp)	T_m (°C)	GenBank accession no.
Rops08	HEX	$(CA)_8TA(CA)_3$	TTCTGAGGAAGGGTTCCGTGG<br>GTTAAAGCAACAGGCACATGG	192–212	63 to 52	AB075033
Rp206	HEX	$(GT)_9$	GCCAAATCCCATTAGATCACAGTTGA<br>AGAAGTTAGACTTACGTGCTGC	200–232	65 to 58	AB353932
Rops05	FAM	$(AC)_2GC(AC)_7$	TGGTGATTAAGTCGCAAG<br>GTGGTTGTGACTTGTACGTAAGTC	114–148	56	AB075031
Rops06	FAM	$(GT)_3ACA(GT)_{11}$	CTAAGGAGGTGCTGACCCTC<br>TTAATCTGTGATGGGACACTG	114–146	65 to 58	AB075032
Rp109	TAMRA	$(AG)_{17}$	GAGGAATCACAAAACCGTTTGG<br>TGGGATTTGAGAGAGTGGTGGTG	119–151	65 to 63	AB353930
Rp200	TAMRA	$(AG)_{23}$	GGTTTCTTTGTTCACCTGCTCTGG<br>ACCTACGTGTCCACGGCTCT	160–185	65 to 60	AB353933

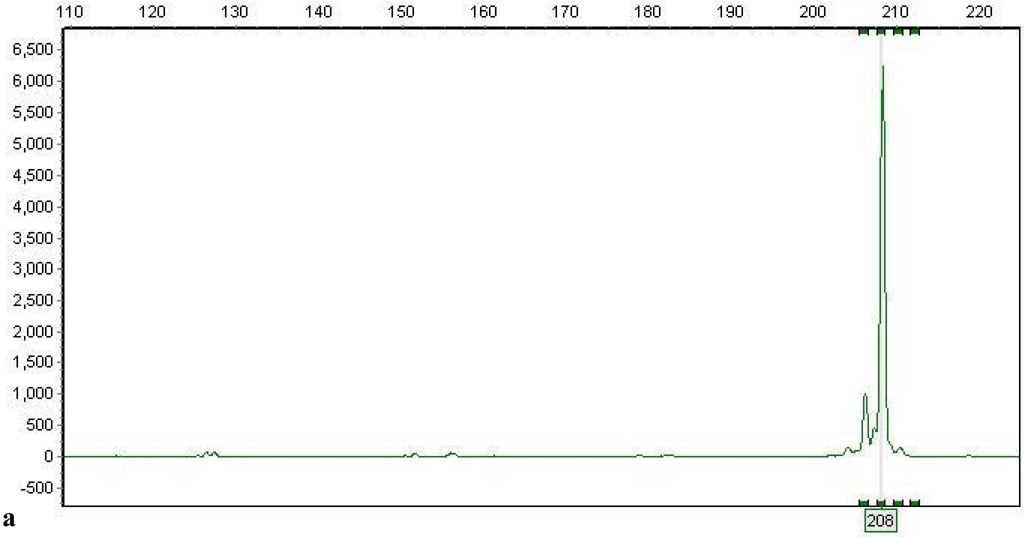

a

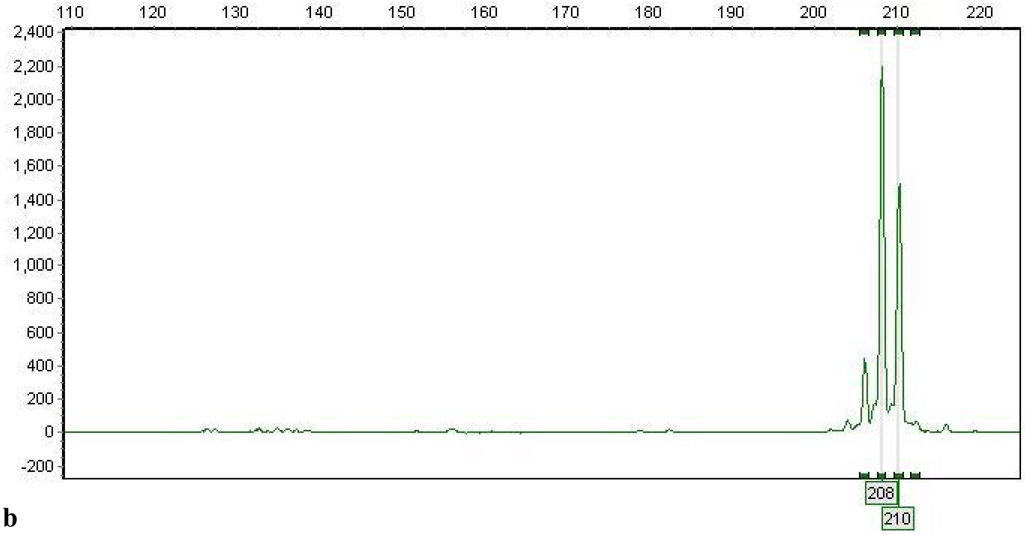

b

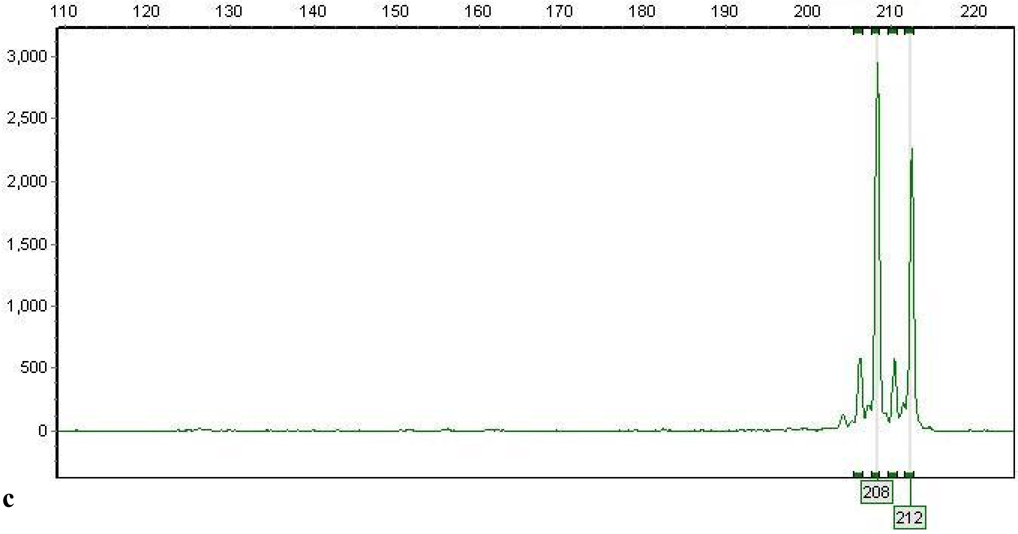

c

Figure S1 An example of the allelic peaks obtained from the maternal tree A and two offspring from locus Rp206. a: maternal tree A; b and c: offspring.
